# MiR-141-3p overexpression suppresses the malignancy of osteosarcoma by targeting FUS to degrade LDHB

**DOI:** 10.1042/BSR20193404

**Published:** 2020-06-10

**Authors:** Lei Wang

**Affiliations:** Department of Orthopedics, First Affiliated Hospital of Chongqing Medical University, No. 1 Youyi Road, Yuzhong District, Chongqing 400016, China

**Keywords:** FUS, LDHB, miR-141-3p, osteosarcoma

## Abstract

Osteosarcoma (OS) is a common malignant bone cancer. Lactate dehydrogenase B (LDHB) has been revealed to act as a tumor promoter in several cancers. It is also revealed to be correlated with poor prognosis in OS, but its molecular mechanism in OS remains veiled. Our work illustrated that LDHB was overexpressed in OS tissues and cells, and it could enhance cell proliferation, migration, and invasion in OS. Subsequently, it was confirmed that fused in sarcoma (FUS) could bind with LDHB to positively regulate the stability of LDHB messenger RNA (mRNA). Besides, FUS expression was revealed to be elevated in OS tissues and positively correlate with LDHB expression. Furthermore, miR-141-3p, down-regulated in OS cells, was identified as the upstream regulator of FUS in OS cells. Besides, miR-141-3p overexpression decreased mRNA and protein levels of FUS and LDHB. More importantly, overexpression of miR-141-3p could impair FUS overexpression-mediated promotion on LDHB mRNA stability and expression. Finally, rescue assays indicated that miR-141-3p regulated OS cells cellular process via regulating LDHB. In sum, miR-141-3p targets FUS to degrade LDHB, thereby attenuating the malignancy of OS cells.

## Introduction

As a malignant bone cancer, osteosarcoma (OS) accounts for over one tenth of all solid tumors [1,2]. Rapid development, high metastasis, and disappointing prognosis are distinct features of OS [3]. Monocytes, macrophages and osteoclasts have been revealed to exert functions in OS [4]. Despite that great progression has been made in therapeutic strategies, there is no remarkable achievement in lowering the mortality of OS patients [5]. Due to the dismal prognosis and the complexity of OS pathogenesis, it is imperative to unveil novel and efficient therapeutic target for OS patients.

High expression of messenger RNA (mRNA) lactate dehydrogenase B (LDHB) has been confirmed to correlate with unfavorable prognosis of OS [6]; hence, the mechanism of LDHB in OS triggers our interest. It has been reported that RNA-binding proteins (RBPs) can regulate RNA stability via interplaying with RNAs within dynamic ribonucleoproteins [7]. Besides, aberrant expression of RBPs was validated to correlate with various diseases, such as metabolic disorders [8], germ-cell development [9], and tumors [10]. Moreover, RBPs could bind with mRNAs, thereby modulating the expression of mRNAs [11]. For example, RBP TAF15 interacted with TRPM2, thereby maintaining TRPM2 mRNA stability [12]. Besides, RBP NELFE aggravated epithelial-to-mesenchymal transition (EMT) in pancreatic cancer by destabilizing NDRG2 mRNA [13]. Furthermore, it was also reported that RBPs could interact with microRNAs (miRNAs) and act as a downstream gene of miRNAs [14]. As reported, miR-301a-3p interacted with RBP FXR1 to degraded p21 in oral cancer [15]. As a subtype of non-coding RNAs, miRNAs are single-stranded and highly conserved with approximately 22 nucleotides in length [16]. Both RBPs and miRNAs have been elucidated to promote or inhibit the occurrence and development of cancer [17,18]. The interaction between RBPs and miRNAs is quite diverse and intriguing.

This work was designed and carried out to identify the molecular mechanism of LDHB in OS. All data supported that overexpression of miR-141-3p restrains fused in sarcoma (FUS)-mediated promotion on mRNA stability of LDHB, thus repressing cell proliferation, migration and invasion in OS.

## Materials and methods

### Tissue samples

56 OS tissues and matched adjacent normal tissues were collected from patients at First Affiliated Hospital of Chongqing Medical University. No patients underwent chemotherapy or radiotherapy before surgery. Written informed consents had been acquired. Right after surgical resection, tissues were frozen using liquid nitrogen and stored at −80°C. The research was approved by the Ethics Committee of First Affiliated Hospital of Chongqing Medical University.

### Cell culture

OS cell lines (U2OS, Saos-2, 143B, MG-63, and HOS) and human osteoblast cell (hFOB) were purchased from American Type Culture Collection (ATCC; Manassas, VA, U.S.A.). Cells were cultivated in DMEM (Corning, Tewksbury, MA, U.S.A.) adding 10% fetal bovine serum (FBS; Gibco, Gaithersburg, MD, U.S.A.) with constant humidity of 5% CO_2_ and 37°C.

### Cell transfection

Specific shRNAs against LDHB (sh-LDHB#1/2) or FUS (sh-FUS#1/2) and NC-shRNAs (sh-NC), along with pcDNA3.1/FUS (OE-FUS), pcDNA3.1/LDHB (OE-LDHB) and the empty vector (OE-NC), were acquired from Genechem (Shanghai, China). Moreover, miR-141-3p mimics and NC mimics were obtained from GenePharma (Shanghai, China). Plasmids were separately transfected into Saos-2 or HOS cells through Lipofectamine 3000 (Invitrogen, Carlsbad, CA, U.S.A.).

### qRT-PCR

Total RNA isolation was undertaken with TRIzol reagent (Invitrogen), and cDNA was later synthesized through the PrimeScript RT reagent kit (Takara, Tokyo, Japan). Thereafter, qRT-PCR was conducted with SYBR Premix Ex Taq (Takara) using CFX96 Real-time PCR system (Bio-Rad, Hercules, CA, U.S.A.). The abundance of target genes was assessed relative to U6 or GAPDH. Relative fold-change was calculated utilizing $2^{-\text{ΔΔ}C_{\text{t}}}$ method.

### CCK-8 assay

Transfected Saos-2 or HOS cells were cultured in 96-well plates (5 × 10^3^ cells/well). Absorbance at 450 nm was read after treatment with CCK-8 reagent (Dojindo, Kumamoto, Japan).

### EdU assay

EdU was conducted with the Cell-Light EdU Apollo488 *in vitro* kit (Ribobio, Shanghai, China). Transfected Saos-2 or HOS cells were planted in 24-well plates (5 × 10^4^ cells/well). Cells were incubated for 2 h with 50 μM EdU. Then, cells were stained in Apollo® fluorescent dye upon fixation in 4% paraformaldehyde (PFA; Sigma-Aldrich, St. Louis, MO, U.S.A.) and permeabilization with 0.5% Triton X-100 (Sigma–Aldrich). After being washed by PBS, cells were counterstained with DAPI (Sigma–Aldrich). Finally, EdU-positive cells were identified with a fluorescence microscope (Olympus, Tokyo, Japan).

### JC-1 assay

Transfected Saos-2 or HOS cells were washed by PBS and subjected to 5 μM JC-1 (Cell Signaling Technology, Beverly, MA, U.S.A.) for 30 min. Changes of mitochondrial membrane potential were assayed with a flow cytometer (Beckman Coulter, Brea, CA, U.S.A.).

### TUNEL assay

Transfected Saos-2 or HOS cells were fixed in ice-cold 2% PFA, and washed using PBS, followed by being stained via the TUNEL kit (Roche, Mannheim, Germany). Following DAPI treatment, TUNEL-positive cells were counted by the fluorescence microscope.

### Transwell invasion assay

Transwell chambers (Corning Costar, Cambridge, MA, U.S.A.) pre-coated with Matrigel (BD Biosciences, Franklin Lakes, NJ, U.S.A.) were applied for invasion assay. Transfected Saos-2 or HOS cells in serum-free medium were plated to the upper chambers. Medium with 10% FBS was put to the lower chambers. 48 h later, invaded cells were fixed in 75% methanol (Sigma–Aldrich) and dyed by crystal violet (Sigma–Aldrich). Assessment of invasive capacity was done through counting invasive cells using a microscope (Olympus).

### Western blot

RIPA buffer (Beyotime, Shanghai, China) was applied to extract protein from transfected Saos-2 or HOS cells. The protein concentration was quantified by BCA kit (Beyotime). Proteins in same amount were loaded on 10% SDS–PAGE gel (Bio-Rad) and shifted to PVDF membranes (Millipore, Bedford, MA, U.S.A.). Following incubation for 1 h in a closed buffer, blots were incubated with primary antibodies against MMP2 (ab97779, Abcam, Cambridge, MA, U.S.A.), MMP7 (ab5706, Abcam), MMP9 (ab38898, Abcam), E-cadherin (ab76319, Abcam), N-cadherin (ab18203, Abcam), Vimentin (ab92547, Abcam), FUS (ab70381, Abcam), LDHB (ab75167, Abcam), and GAPDH (ab9485, Abcam). Later, secondary antibodies were added and the ECL PLUS/KIT (GE Healthcare, Milwaukee, WI, U.S.A.) was applied for chemiluminescence detection.

### RNA pull down

Cell lysates of Saos-2 or HOS cells were incubated with biotin labeled-RNA probes for LDHB and FUS, and no-biotin probe was taken as control, separately. After adding streptavidin magnetic beads (Invitrogen), RNA complex was tested via qRT-PCR.

### Subcellular fractionation

Cytoplasmic and Nuclear RNA Purification Kit (Norgen, Ontario, Canada) was utilized in line with the instructions for the separation and purification of the cytoplasmic and nuclear RNA. qRT-PCR determined the expression of FUS. GAPDH served as cytoplasmic control and U6 acted as nuclear control.

### Luciferase reporter assay

The sequences of LDHB promoter were sub-cloned into pGL3-luciferase vector (Promega, Madison, WI, U.S.A.) and co-transfected into Saos-2 or HOS cells with miR-141-3p mimics or NC mimics. The pmirGLO vectors (Promega) containing wild-type or mutant sequences of miR-141-3p in FUS 3′-UTR were co-transfected with miR-141-3p mimics or NC mimics. Relative luciferase activity was obtained via dual-luciferase reporter assay system (Promega).

### Actinomycin D treatment

For blocking mRNA transcription, transfected Saos-2 or HOS cells were mixed with 2 mg/ml actinomycin D (ActD; Sigma–Aldrich) for 0, 3, 6, and 9 h. LDHB mRNA level was revealed by qRT-PCR and normalized to the values measured in 0 h.

### Statistical analysis

Values from three independent assays were imported into SPSS 22.0 (IBM, Chicago, IL, U.S.A.) and shown as means ± SD. Pearson’s correlation analysis was applied for assessing the expression correlation between genes. Differences in groups were identified with Student’s *t*-test or one-way ANOVA, with *P*<0.05 as standard.

## Results

### LDHB is overexpressed in OS and enhances OS malignancy

**Figure 1:**
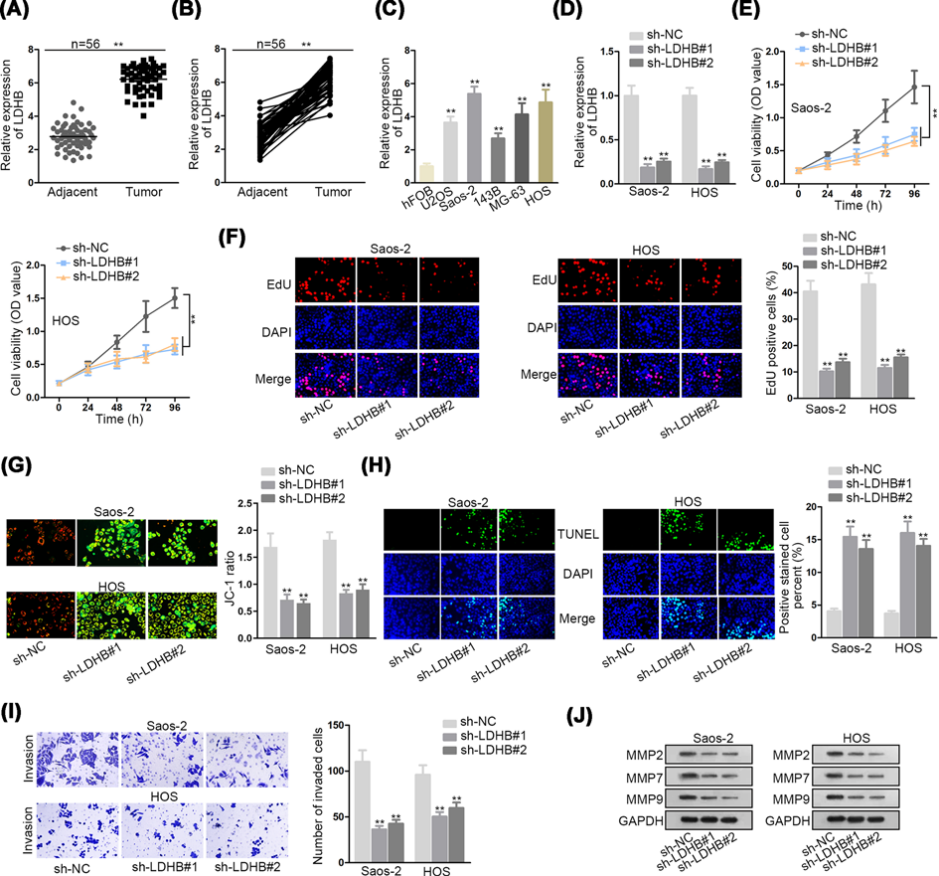
FLDHB is overexpressed in OS and enhances OS malignancy (**A,B**) The expression of LDHB was detected by qRT-PCR in OS tissues and paired adjacent normal tissues. (**C**) LDHB levels in OS cells (U2OS, Saos-2, 143B, MG-63, HOS) and hFOB were detected by qRT-PCR. (**D**) The knockdown efficiency of LDHB in Saos-2 and HOS cells was verified by qRT-PCR. (**E,F**) CCK-8 and EdU assays were applied to measure cell proliferation in sh-NC or sh-LDHB#1/2-transfected cells. (**G,H**) The effect of LDHB depletion on cell apoptosis were researched by JC-1 and TUNEL assays. (**I**) The invasion in sh-LDHB#1/2-transfected cell was observed in transwell assay. (**J**) Expressions of migration-related proteins (MMP2, MMP7 and MMP9) in sh-LDHB#1/2-transfected cells were detected by Western blot analysis. **P<0.01.

High expression of LDHB has been suggested to predict an unfavorable prognosis in OS [6]. In our study, the expression of LDHB in OS was detected. To begin with, qRT-PCR analysis suggested that LDHB level was considerably elevated in OS tissues compared with paired adjacent normal tissues (Figure 1A,B). In addition, LDHB expression was much higher in OS cells (U2OS, Saos-2, 143B, MG-63, HOS) than in human fetal osteoblast (hFOB) (Figure 1C). Owing to the up-regulation of LDHB in OS, we carried out loss-of-function assay to explore biological effect of LDHB on OS cell growth and invasion. As shown in Figure 1D, LDHB level was significantly attenuated in Saos-2 and HOS cells transfected with sh-LDHB#1/2. Subsequently, CCK-8 assay revealed that LDHB depletion induced a repression in OS cell proliferation (Figure 1E). Then, EdU assay also confirmed the suppressive effect of LDHB inhibition on OS cell proliferation (Figure 1F). Furthermore, JC-1 assay indicated that the apoptosis was promoted in sh-LDHB#1/2-transfected OS cells (Figure 1G). The same result was observed in TUNEL assay that LDHB knockdown enhanced cell apoptosis in OS (Figure 1H). Moreover, transwell assay demonstrated that the number of invaded cells was decreased by LDHB deficiency in OS (Figure 1I). Finally, we detected the levels of migration-relevant proteins (MMP2, MMP7, MMP9) and EMT-relevant proteins (E-cadherin, N-cadherin) upon LDHB depletion. Western blot assay indicated that the protein levels of MMP2, MMP7, and MMP9 were reduced by LDHB knockdown (Figure 1J), indicating that cell migration and invasion was interfered after LDHB was silenced. Importantly, EMT-related proteins were detected and we found that the protein levels of N-cadherin and Vimentin were reduced by LDHB knockdown, while the protein level of E-cadherin was increased (Supplementary Figure S1A), indicating that silencing of LDHB hampered EMT process. In conclusion, LDHB expression is up-regulated in OS tissues and cells, and promotes cell proliferation, migration, and invasion.

### FUS promotes the stability of LDHB mRNA in OS cells

**Figure 2:**
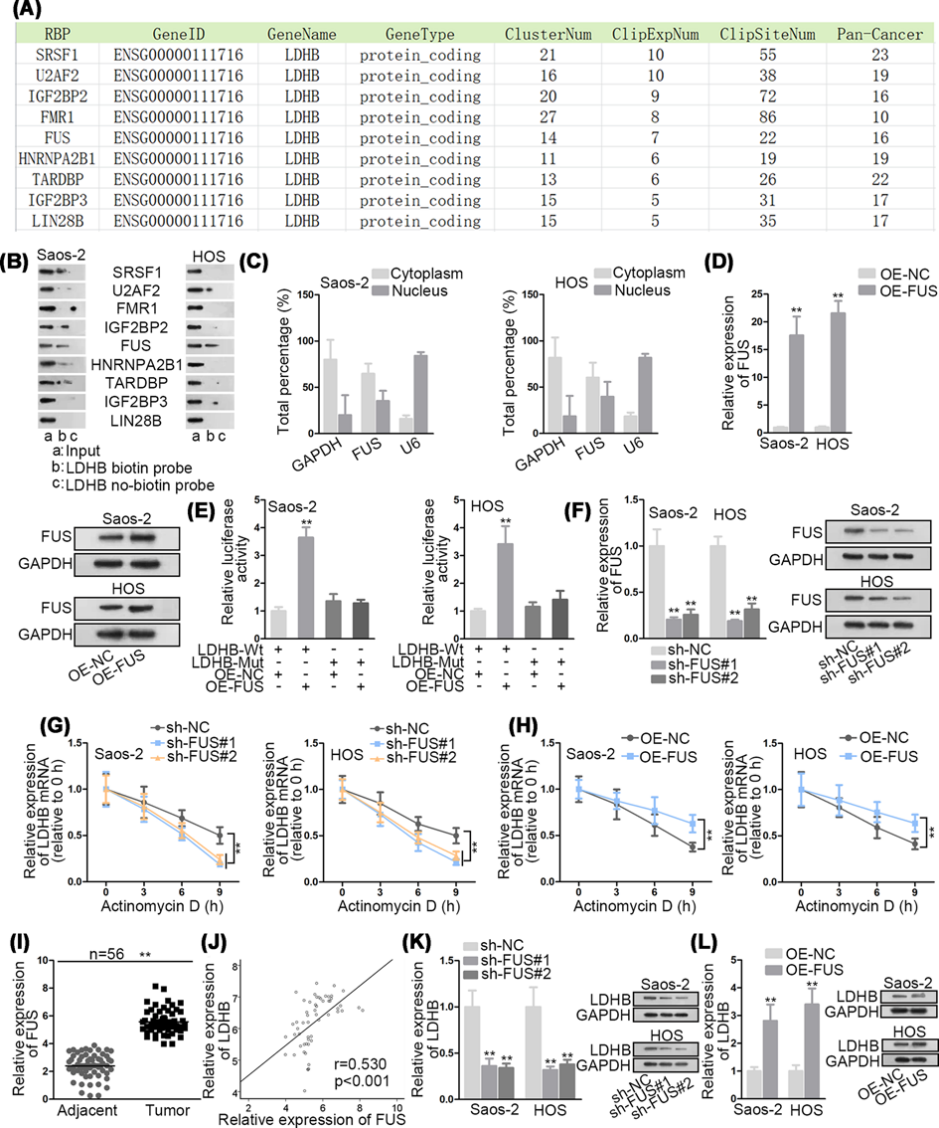
FUS promotes the stability of LDHB mRNA in OS cells (**A**) The possible RBPs for LDHB were screened out by starBase. (**B**) The binding between LDHB and nine candidate RBPs was verified by RNA pull down assay. (**C**) The localization of FUS was determined by subcellular fractionation assay. (**D**) The overexpression efficiency of FUS in Saos-2 and HOS cells was measured by qRT-PCR and Western blot analyses. (**E**) Luciferase reporter assay was carried out to investigate the combination between FUS and LDHB. (**F**) qRT-PCR and Western blot analyses examined the interference efficiency of FUS in Saos-2 and HOS cells. (**G,H**) The effect of FUS down-regulation or up-regulation on LDHB mRNA stability after ActD treatment was explored by qRT-PCR. (**I**) The expression of FUS in OS tissues and paired adjacent normal tissues was detected by qRT-PCR. (**J**) The expression correlation between FUS and LDHB in OS tissues was analyzed by Pearson’s correlation analysis. (**K,L**) The influence of FUS depletion or overexpression on LDHB mRNA and protein levels was assessed by qRT-PCR and Western blot analyses. **P<0.01.

It has been established that the stability of mRNAs could be enhanced or weakened by RBPs [19]. Thus, we explored whether LDHB mRNA could be stabilized by a specific RBP. It was searched from starBase (http://starbase.sysu.edu.cn/index.php) that there were nine potential RBPs for LDHB (Figure 2A). To further filtration, RNA pull down assay was performed and results indicated that FUS was abundantly pulled down by LDHB biotin probe, while other RBPs did not show a significant enrichment (Figure 2B). It has been reported that RBPs are critical regulators involved in post-transcriptional regulation [20]. To confirm whether FUS regulate LDHB expression at post-transcriptional level in the cytoplasm, subcellular fractionation assay was carried out. The results disclosed that the cytoplasmic part of Saos-2 and HOS cells contained higher FUS expression (Figure 2C). Therefore, we hypothesized that FUS might act as a RBP for LDHB. Subsequently, FUS was overexpressed in Saos-2 and HOS cells for the following luciferase reporter assay. The results of qRT-PCR suggested that the mRNA and protein levels of FUS were both evidently elevated by pcDNA3.1/FUS (Figure 2D). As displayed in Figure 2E, the luciferase activity of LDHB-Wt reporter was increased by FUS up-regulation in Saos-2 and HOS cells. Afterwards, qRT-PCR analyzed that the transfection of sh-FUS#1/2 triggered a notable decrease in FUS expression (Figure 2F). As exhibited in Figure 2G,H, with ActD treatment, we observed that LDHB mRNA stability was weakened by FUS inhibition and promoted by FUS up-regulation compared with control group. Moreover, FUS expression was revealed to be up-regulated in OS tissues and positively correlate with LDHB expression (Figure 2I,J). Finally, qRT-PCR and Western blot analyses displayed that LDHB mRNA and protein levels were reduced by FUS knockdown and increased by FUS overexpression (Figure 2K,L). In sum, FUS stabilizes LDHB mRNA in OS cells.

### MiR-141-3p targets FUS to influence the expression of LDHB

**Figure 3:**
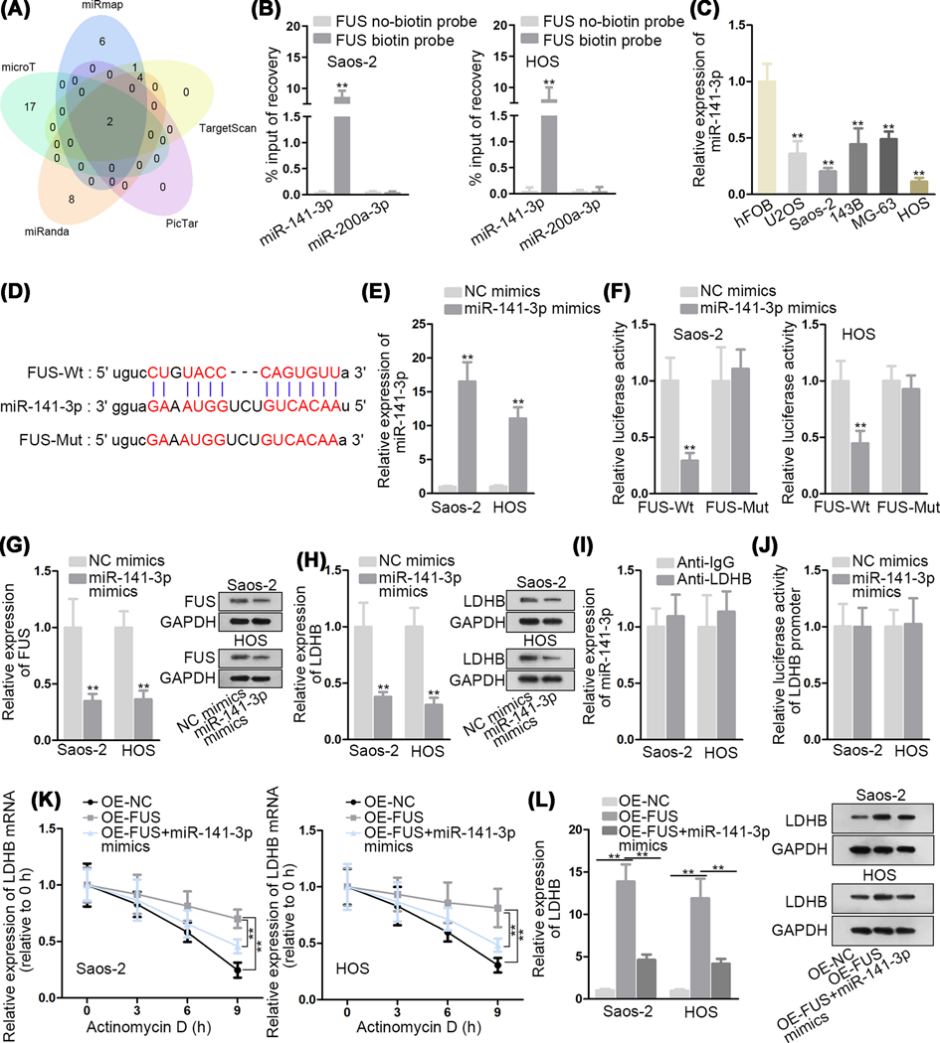
MiR-141-3p targets FUS to influence the expression of LDHB (**A**) The potential miRNAs targeting FUS were spot via starBase. (**B**) RNA pull down assay was performed to research the binding between FUS and two candidate miRNAs. (**C**) The expression of miR-141-3p in OS cells was detected by qRT-PCR. (**D**) The underlying binding site between miR-141-3p and FUS was probed via starBase. (**E**) The overexpression efficiency of miR-141-3p in Saos-2 and HOS cells was affirmed by qRT-PCR. (**F**) Luciferase reporter assay was carried out to confirm the binding between miR-141-3p and FUS. (**G,H**) The mRNA and protein level of FUS and LDHB in miR-141-3p mimics-transfected cells was examined by qRT-PCR and Western blot analyses. (**I**) The interaction between miR-141-3p and LDHB was estimated by RIP assay. (**J**) The binding between miR-141-3p and LDHB promoter was investigated by luciferase reporter assay. (**K**) The influence of miR-141-3p overexpression on FUS up-regulation-mediated LDHB mRNA stability was evaluated by qRT-PCR. (**L**) qRT-PCR and Western blot analyses were carried out to reflect LDHB expression in pcDNA3.1/FUS or pcDNA3.1/FUS+miR-141-3p mimics-transfected cells. **P<0.01.

It has been reported that RBPs could interact with miRNAs and regulated by miRNAs at post-transcriptional level [15]. Therefore, we explored the upstream mechanism of FUS and wondered whether FUS could combine with certain miRNAs. Through starBase, two miRNAs (miR-141-3p, miR-200a-3p) that could target FUS were screened out (Figure 3A). RNA pull down assay showed that miR-141-3p rather than miR-200a-3p was pulled down by FUS biotin probe (Figure 3B). Hence, miR-141-3p was chosen for further investigation. As shown in Figure 3C, miR-141-3p level was down-regulated in OS cells. Besides, the potential binding site between miR-141-3p and FUS was predicted from starBase (Figure 3D). Then, miR-141-3p was overexpressed by miR-141-3p mimics for following exploration. qRT-PCR showed that miR-141-3p mimics raised miR-141-3p level in Saos-2 and HOS cells (Figure 3E). And the decreased luciferase activity of FUS-Wt reporter in miR-141-3p mimics-transfected cells proved the affinity between miR-141-3p and FUS (Figure 3F). Additionally, it was also reflected by qRT-PCR and Western blot analyses that miR-141-3p overexpression caused an inhibition in both FUS and LDHB expressions at mRNA and protein levels (Figure 3G,H). Subsequently, RIP assay expounded that miR-141-3p could not bind with LDHB (Figure 3I). More importantly, the luciferase activity of LDHB promoter was not affected by miR-141-3p mimics (Figure 3J). Through ActD treatment, we found that LDHB mRNA stability strengthened by FUS up-regulation was largely decreased by miR-141-3p overexpression (Figure 3K). Finally, the elevated LDHB mRNA and protein expressions in FUS overexpressed cells were impaired by miR-141-3p up-regulation (Figure 3L). In brief, miR-141-3p targets FUS to influence the expression of LDHB.

### MiR-141-3p inhibits OS proliferation, migration, and invasion in LDHB-dependent manner

**Figure 4:**
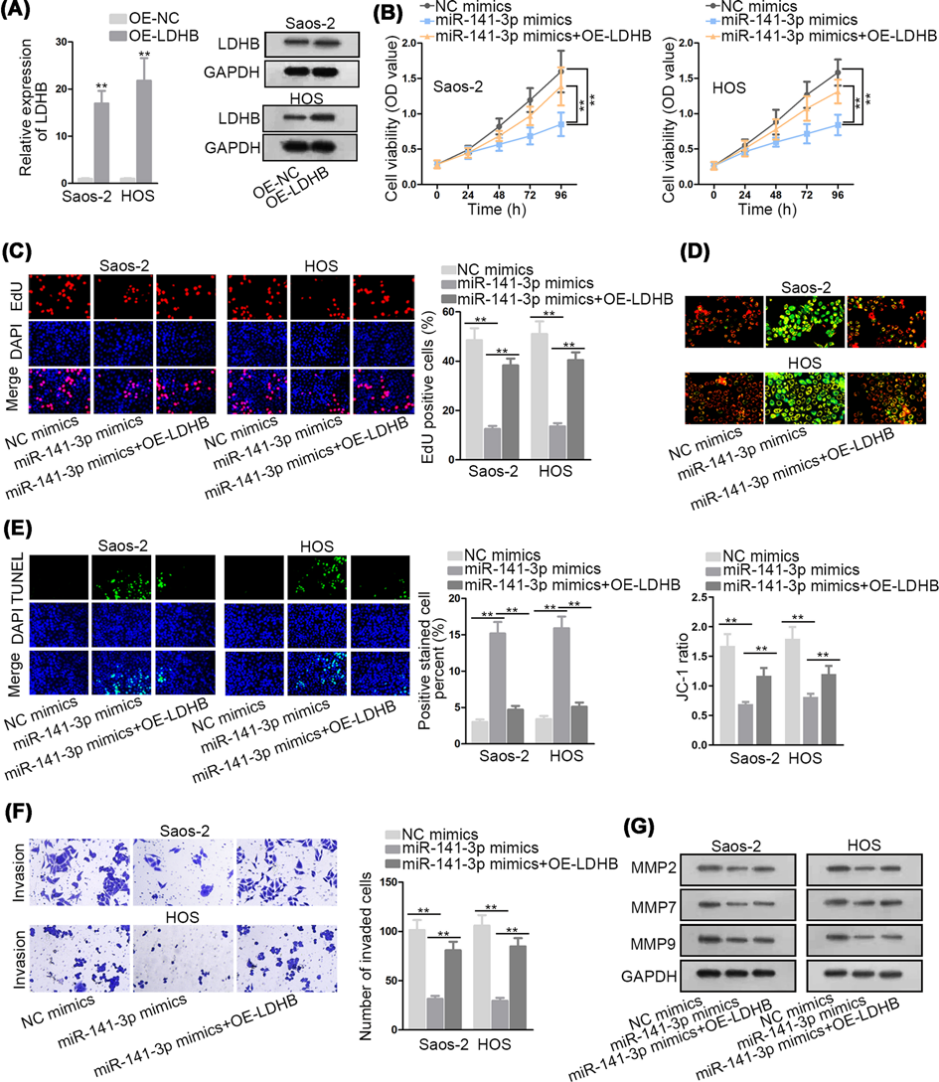
MiR-141-3p inhibits OS proliferation, migration and invasion in LDHB-dependent manner (**A**) The overexpression efficiency of LDHB in Saos-2 and HOS cells was confirmed by qRT-PCR and Western blot analyses. (**B,C**) Cell proliferative ability in indicated transfected cells was reflected by CCK-8 and EdU assays. (**D,E**) JC-1 and TUNEL assays were performed to detect cell apoptosis in each group. (**F**) Cell invasive ability in indicated transfected cell was evaluated by transwell assay. (**G**) Western blot assay measured expressions of migration-related proteins in each group. **P<0.01.

Finally, we conducted rescue assays to clarify whether miR-141-3p regulated OS development by mediating LDHB. First, LDHB was overexpressed in Saos-2 and HOS cells by transfecting pcDNA3.1/LDHB (Figure 4A). Then, CCK-8 and EdU assays reflected that the suppressed cell proliferation in miR-141-3p mimics-transfected cells was abrogated by LDHB overexpression (Figure 4B,C). Furthermore, it was examined by JC-1 and TUNEL assays that the promotion in cell apoptosis triggered by miR-141-3p overexpression was countervailed by LDHB up-regulation (Figure 4D,E). Finally, transwell assay and Western blot analysis illustrated LDHB overexpression abolished miR-141-3p up-regulation-mediated inhibition on cell migration and invasion (Figure 4F,G). In a word, miR-141-3p hampers OS progression via regulating LDHB.

## Discussion

As an important glycolytic enzyme, LDHB has been reported to be overexpressed in triple-negative breast cancer [21] and promote the tumorigenesis of colorectal cancer [22]. Although LDHB has been confirmed to express at a high level in OS cells [23], the functional role and molecular mechanism of LDHB have not been researched in OS. This work also displayed high LDHB expression in OS tissues and cells. Moreover, LDHB was proved to exert oncogenic function by promoting cell growth and migration in OS cells. In sum, LDHB is a tumor promoter in OS.

It has been well established that RBPs could exert their functions via binding with specific RNA in a few processes, including transcription, translation, and degradation [24]. It has been uncovered that RBP PUM2 competed with miRNAs to bind with STARD13 3′UTR, thus inhibiting cell growth and migration in OS [25]. Due to the high level of LDHB in OS, we hypothesized that LDHB was stabilized by certain RBP(s). Combined with results from starBase and our experiments, FUS was identified as the RBP for LDHB. RBP FUS has been discovered to act as a tumor enhancer in several cancers such as thyroid cancer, non-small cell lung cancer and glioma [26–28]. Apart from the verified affinity between FUS and LDHB, it was also revealed that FUS could elevate the stability of LDHB mRNA. More importantly, it was detected that FUS was markedly overexpressed in OS tissues, and it could positively regulate LDHB mRNA and protein levels. These data suggested that FUS could interact with LDHB to stabilize LDHB mRNA.

Extensive literatures have testified that RBPs could interacted with miRNAs [15]. MiR-141-3p has been unearthed to serve as an anti-cancerous gene in cancers such as gastric cancer and hepatocellular carcinoma [29,30]. Besides, existing researches also pointed out that miR-141-3p was down-regulated in OS tissues and could suppress OS cell proliferation [31,32]. In our paper, miR-141-3p was remarkably down-regulated in OS cells, and its interaction with FUS was certified. More importantly, miR-141-3p overexpression could reduce the expressions of FUS and LDHB at mRNA and protein levels. Furthermore, it was confirmed that miR-141-3p could neither bind with LDHB nor transcriptionally activate LDHB. However, miR-141-3p overexpression could impede LDHB mRNA stability and expression which enhanced by FUS up-regulation. Rescue assays illustrated that miR-141-3p inhibited OS malignancy by regulating LDHB.

In conclusion, miR-141-3p was down-regulated in OS cells and suppressed the malignancy of OS by regulating FUS-mediated LDHB. The present paper provides a novel insight into figuring out the pathology of OS.

## Supplementary Material

Supplementary Figure S1

## Abbreviations

EMT: epithelial-to-mesenchymal transition
FBS: fetal bovine serum
FUS: fused in sarcoma
hFOB: human fetal osteoblast
LDHB: lactate dehydrogenase B
mRNA: messenger RNA
OS: osteosarcoma
PFA: paraformaldehyde
RBP: RNA-binding protein
