# Supplementary material for: MiR-141-3p overexpression suppresses the malignancy of osteosarcoma by targeting FUS to degrade LDHB
Source: Biosci Rep. 2020 Jun 10;40(6):BSR20193404. doi: 10.1042/BSR20193404 (PMC7286874; doi:10.1042/BSR20193404)
Supplement: Supplementary Figure S1 [file BSR-2019-3404_supp.pdf]

Figure S1

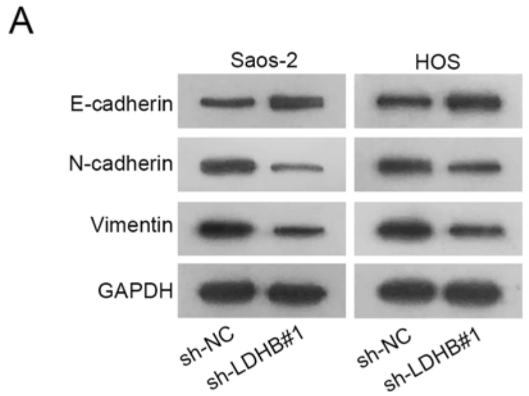

Figure S1 The effect of LDHB silence on EMT process

(A) Western blot assay measured expressions of EMT-related proteins in each group.
